# Polydatin inhibits hepatocellular carcinoma via the AKT/STAT3-FOXO1 signaling pathway

**DOI:** 10.3892/ol.2019.10856

**Published:** 2019-09-12

**Authors:** Jian Jiang, Yaodong Chen, Tianxiu Dong, Minlu Yue, Yu Zhang, Tingting An, Jiuwei Zhang, Pengfei Liu, Xiuhua Yang

Oncol Lett 17: 4505-4513, 2019; DOI: 10.3892/ol.2019.10123

Subsequently to the publication of this article, the authors have realized that Fig. 3 contained a duplicated panel: In Fig. 3A, the data correctly shown for the 0 h, ‘Control’ panel was inadvertently copied across to the 0 h, ‘100 µmol/l’ panel. The revised version of Fig. 3, featuring the correct data for the 0 h, ‘100 µmol/l’ panel, is shown opposite.

Note that this error did not affect the overall conclusions reported in the paper. The authors apologize to the Editor of *Oncology Letters* and to readership for any inconvenience caused.

## Figures and Tables

**Figure 3.**
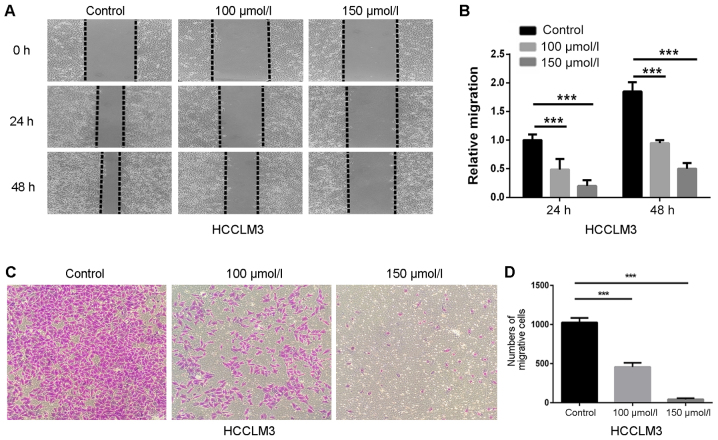
Polydatin inhibits migration of HCCLM3 cells. (A) Suppressive effects of polydatin on migration were assessed via wound healing assay (magnification, ×200). (B) Statistical analysis of the relative distances of cell migration. (C) Migration assay of HCCLM3 cells following treatment with polydatin at non-cytotoxic concentrations (0–150 μmol/l) (magnification, ×200). (D) Statistical analysis of the number of cells crossing the membrane. ^***^P<0.001 vs. control.

